# Predictors of Increased Physical Activity in the Active for Life Program

**Published:** 2008-12-15

**Authors:** Sara Wilcox, Marsha Dowda, Andrea Dunn, Marcia G. Ory, Carol Rheaume, Abby C. King

**Affiliations:** Department of Exercise Science, Arnold School of Public Health; Department of Exercise Science, University of South Carolina, Columbia, South Carolina; Klein-Buendel, Golden, Colorado; Texas A&M Health Science Center, College Station, Texas; Prevention Research Center, University of South Carolina, Columbia, South Carolina; Stanford Prevention Research Center and Stanford School of Medicine, Stanford, California

## Abstract

**Introduction:**

Targeting of evidence-based programs can be improved by knowing who benefits least and most. We examined pretest predictors of increased physical activity among participants enrolled in Active for Life.

**Methods:**

Participants (N = 1,963) from 9 community-based organizations took part in a 6-month telephone-based or a 20-week group-based behavioral physical activity program and completed a pretest survey; 1,335 participants returned posttest surveys. Interactions tested whether increases in physical activity differed over time, according to baseline characteristics.

**Results:**

In the telephone-based program, participants who were younger and less active at pretest and those who had higher pretest social support showed greater intervention effects. In the group-based program, younger participants, those less active at pretest, women, Hispanics/Latinos, heavier participants, and those who reported more health conditions and osteoporosis showed greater intervention effects.

**Conclusion:**

Participant response to the 2 programs varied by age, baseline activity level, and other factors. For 6 of the 8 variables associated with differential outcomes, the least active group improved the most, which suggests that the programs worked especially well for participants most in need. Participants who were older than 75 years (both groups) and those who reported lower physical activity social support (in the telephone-based program) on entry did not respond as well and may require alternative or more intensive intervention strategies.

## Introduction

Most older adults (aged 65 and older) have a chronic health condition, and 50% have 2 or more (1). By 2030 a 25% increase in health care expenditures is expected because of the population increase in older adults (1). Many age-related chronic health conditions are caused by lifestyle factors. Physical activity can reduce disease burden and disability and enhance quality of life in older adults (2), but physical activity level declines with age (3,4).

In the previous 2 decades, more has been learned about how to effectively increase participation in physical activity. Reviews of physical activity interventions with older adults report favorable outcomes for interventions that use behavioral strategies and theories and comparable outcomes for supervised home-based and class or group formats (5-8). However, little is known about differences in response to interventions. Furthermore, few programs deemed effective in randomized trials are disseminated to community settings (9). We know little about how these interventions might work in real-world settings (10). Population-level changes in physical activity are likely to occur only when effective interventions are translated for widespread use in community settings (11).

The Active for Life (AFL) initiative examines the translation of 2 efficacious, theory-based (12-14) physical activity programs to community settings (15,16). The theories used are Social Cognitive Theory and the Transtheoretical Model. Social Cognitive Theory (12) emphasizes the reciprocal interactions between the person, environment, and behavior. Key intervention components of this theory include increasing self-efficacy or confidence in overcoming barriers to behavior change and enhancing self-regulatory skills such as goal-setting, self-monitoring, problem-solving, and self-reward. The Transtheoretical Model (14) posits that people make changes gradually and in stages and that a person's readiness for behavioral change should be used to guide the types of intervention strategies delivered. A previous study demonstrated the effective translation of the interventions tested in AFL into community settings with an effect size similar to that of the original efficacy studies but with a more representative sample (15,16). Our purpose was to examine whether intervention effects for these 2 programs differed by the following pretest characteristics of the sample: demographic factors, health-related variables, psychosocial characteristics, and initial physical activity levels. AFL's size and sample diversity allow for these types of predictor analyses. Knowing the characteristics of participants who benefit most and least from an intervention has programmatic implications (17,18). Understanding differential predictors can also help match people with intervention or treatment options (19).

## Methods

### Program overview

AFL is a 4-year initiative, described in detail elsewhere (15,16) (www.activeforlife.info), that evaluated the 2 evidence-based behavioral programs we studied. As implemented in AFL, Active Choices (AC) is a 6-month program developed by Stanford University and delivered through a face-to-face orientation followed by up to 8 one-on-one telephone counseling calls (20-23). Active Living Every Day (ALED), developed by The Cooper Institute and Human Kinetics, Inc, is a 20-week program delivered in small groups (24,25). Participants meet weekly and are encouraged to provide support and share successes and challenges. Nine lead organizations at 12 sites were funded to participate in AFL (Table 1) (15,16).

### Participants

During the entire AFL initiative, each lead organization was expected to recruit 900 participants for a study total of 8,100. Recruitment strategies were tailored by sites to their communities and targeted adults aged 50 years and older. All sites used the same screening instruments and enrolled those who were underactive (engaged in physical activity ≤2 days per week and <120 minutes per week) and free of serious medical conditions or disabilities that required higher levels of supervision on the basis of the site's individualized risk management plan, as described elsewhere (15,16). Although the revised Physical Activity Readiness Questionnaire (PAR-Q) was administered at each site, only 2 sites required medical clearance in response to a positive PAR-Q. All physical activity participants with a positive PAR-Q, however, were encouraged to discuss physical activity with their health care provider.

### Design and procedure

Comparable comprehensive preprogram and postprogram surveys were administered to all year 1 participants (approximately 100 per site) and to the first 100 participants per site in years 3 and 4. Comprehensive surveys were administered only to the first 100 participants in the later years because we deemed this number to be an adequate sample size for detecting change over time and because it reduced site burden. We report data for participants who completed the comprehensive surveys in years 1 and 3. Data for participants in years 2 and 4 are not included. Surveys were not collected in year 2, and adaptations to the original ALED program model were tested in year 4.

All participants completed an informed consent form approved by the institutional review boards of the 2 participating universities (an evaluation team and the national program office) and by the review boards or legal departments of the 9 lead organizations. Participants completed a brief demographic questionnaire and were given the pretest survey and a postage-provided envelope addressed to the evaluation team. For ALED, posttest surveys were sent to the site and administered in 1 of the 2 last sessions or they were sent directly to participants 2 weeks before completion of their program. For AC, all posttest surveys were sent directly to participants 2 weeks before completion of their program. Postage-paid envelopes addressed to the evaluation team were included. Each participant who returned a survey entered a drawing for a $20 gift card to a local retail store (a 1 in 25 chance). Because of input from a local oversight board, 1 AC site did not participate in the gift card incentive beyond the first year.

### Measures

We collected data on age, sex, race, Latino ethnicity, and years of education. Participants self-reported height and weight to compute body mass index (BMI) (26), rated their health from poor to excellent, and indicated whether they had ever been told by a health professional they had diabetes, hypertension, arthritis, coronary heart disease (ie, self-report of angina, coronary heart disease, or a heart attack), or osteoporosis (27).

Physical activity self-efficacy was measured with a 5-item scale in which participants rated their confidence in being able to be regularly physically active when faced with common barriers (α = .87) (28). Social support from friends and family was measured with the 5-item scale (each with a 4-point response scale) developed for the US Women's Determinants Study (29), which used questions derived from the commonly used but significantly longer scale developed by Sallis et al (α = .70) (30). Participants also completed the widely used 10-item Center for Epidemiological Studies Depression Scale (31-33) by rating the frequency with which they experienced symptoms of depression during the past week (α = .82). Finally, participants completed the 4-item version of the Perceived Stress Scale (34,35), a briefer form of an extensively used questionnaire that was designed to measure the degree to which situations in one's life are appraised as stressful (α = .69). All psychosocial variables were calculated as continuous variables and were also categorized into tertiles.

The Community Healthy Activities Model Program for Seniors (CHAMPS) questionnaire, a 41-item self-report measure of physical activity, was the primary outcome measure (36). It includes activities of all intensity levels typically undertaken by older adults for exercise, recreation, and daily living. The CHAMPS questionnaire has strong psychometric properties, including demonstrated validity (37), test-retest reliability (37), and sensitivity to change (22,23,36,38,39). We derived the minutes per week spent in moderate-intensity and vigorous-intensity physical activity (MVPA). Physical activity level was also categorized into tertiles. We used a secondary 3-item measure from the Behavioral Risk Factor Surveillance System (BRFSS) (27) to assess participation, frequency, and duration of moderate-intensity physical activity to classify participants as sedentary, underactive, or regularly active (40).

### Statistical analyses

We conducted separate analyses for AC and ALED because the programs differed in length, mode of delivery, and characteristics of participants. Primary analyses examined whether changes from pretest to posttest in MVPA hours per week (as reported on the CHAMPS questionnaire) differed by pretest predictors (ie, time x predictor interactions). We conducted a separate repeated-measures analysis of covariance that tested each time x predictor interaction. In analyses that did not include the variables of sex, race/ethnicity, education, health rating, and BMI, these variables were entered as covariates because of their known association with MVPA. Site clustering was accounted for by using SAS version 9 (SAS Institute Inc, Cary, North Carolina). MVPA was positively skewed at pretest and somewhat skewed at posttest but was normalized with a square-root transformation.

We conducted 2 additional sets of analyses to better understand each potential predictor variable, consistent with the approach recommended elsewhere (18). Statistical significance was set at *P* < .05. First, we examined the percentage who met recommendations of the Centers for Disease Control and Prevention (CDC)-American College of Sports Medicine (ACSM) (determined by using the BRFSS physical activity questions) in association with each predictor variable at pretest and posttest, controlling for the same covariates as in the primary analyses. We then tested whether the percentage meeting recommendations changed differentially by each predictor variable over time (time x predictor interaction), controlling for covariates.

## Results

### Description of the sample

A total of 841 participants in year 1 and another 1,122 participants in year 3 completed pretest surveys. Of these, 72 participants in year 1 (8.6%) and 137  participants in year 3 (12.2%) withdrew from the program or the program and evaluation. Posttest surveys were returned by 613 participants in year 1 (72.9%) and 730 participants in year 3 (65.1%). Eight participants were excluded from all analyses because of unusable data for the primary outcome. The final sample was 1,955 (881 AC and 1,074 ALED participants;  Table 2).

The following sample characteristics were associated with lower postsurvey response rates: younger age, nonwhite race/ethnicity, lower educational attainment, not being married or partnered, having diabetes or coronary heart disease, poorer self-rated health, higher physical activity social support, lower physical activity self-efficacy, higher depressive symptoms, higher perceived stress, and lower physical activity level (data not shown). We found no statistically significant differences for sex, BMI, number of health conditions, or the presence of hypertension, arthritis, stroke, or osteoporosis.

### Predictors of physical activity

We calculated adjusted square-root minutes per week of MVPA at pretest and posttest for each level of the predictor variable, effect sizes, and *P* values for the change analyses (Table 3). For AC, younger participants and those with higher pretest social support and physical activity showed greater increases in physical activity (all *P* values <.05). Hispanic/Latino participants, those with fewer health conditions, and those without coronary heart disease were also more likely to show greater increases in physical activity, although these interactions did not reach significance (*P* < .10).

For ALED, younger participants, women, Hispanic/Latino participants, those with higher pretest BMI and more health conditions, those reporting osteoporosis, and those reporting lower pretest physical activity showed greater increases in physical activity (all *P *values < .05). ALED participants with more than a high school education and those with hypertension were also more likely to increase physical activity, but these interactions did not reach significance (*P* < .10).

### Percentage meeting CDC-ACSM recommendations

We determined the percentage of participants who met CDC-ACSM recommendations at pretest and posttest (for categorical variables) and odds ratios and 95% confidence intervals (for all variables) (Table 4 and Table 5). Because the program targeted underactive and sedentary participants, only posttest findings are described here. For AC, participants who had fewer pretest health conditions, were free of arthritis and coronary heart disease, had more positive self-rated health at pretest, and reported higher pretest physical activity were significantly more likely to meet physical activity recommendations at posttest. For ALED, participants who had higher levels of education, higher pretest social support, higher pretest self-efficacy, lower pretest perceived stress, and higher pretest physical activity were significantly more likely to meet physical activity recommendations at posttest.

The next set of analyses examined whether the change in the percentage meeting CDC-ACSM recommendations from pretest to posttest (as measured by BRFSS physical activity questions) differed by each predictor variable after controlling for potential confounders (ie, time x pretest predictor interactions). For AC, significant time x pretest predictor interactions indicated that younger participants (*P* = .03), those with fewer health conditions (*P* = .03), and those without coronary heart disease (*P* = .005) showed the largest increases in intervention effects (data not shown.) For ALED, time x pretest predictor interactions indicated that younger participants (*P* = .03), those with higher levels of education (*P* = .05), those with higher BMIs (*P* = .003), and those with lower physical activity levels at pretest (as measured by the CHAMPS questionnaire) (*P* = .007) showed the largest intervention effects (data not shown). No other interactions reached significance.

## Discussion

Although examining data to determine which population segments do better or worse with behavioral interventions (19) is critical, few studies have adequate sample sizes and diversity to allow such analyses. AFL recruited a large sample of midlife and older adults that showed diversity in demographic, health, and psychosocial characteristics. Seventeen baseline variables were examined as potential predictors of change in physical activity. Eight variables predicted differential outcomes in the primary analyses; of these, 6 groups that were initially less active showed larger increases in physical activity. These results are encouraging and suggest that when the 2 behaviorally oriented physical activity programs were implemented in real-world settings, most midlife and older adults responded favorably to both, and no groups were adversely affected.

Primary analyses based on the CHAMPS questionnaire revealed that most participants achieved similar levels of posttest physical activity. Several groups that were initially less active showed larger intervention gains over time. Thus, the interventions worked best in those for whom they were designed, the groups that were initially less active. We noted 2 exceptions to this pattern. Participants in the oldest age group (AC and ALED) and those with lower levels of pretest social support (AC) showed significant and substantial but smaller increases in physical activity than did their counterparts.

BRFSS analyses indicated relatively few pretest differences. We found a substantial number of posttest differences, however, that were generally consistent with the literature on the correlates of physical activity (41). Despite significant posttest differences, all groups made substantial improvements over time. Furthermore, no groups were harmed by either intervention, which is a very important finding given the limited exclusion criteria and large sample (19).

The pattern of results differed for the 2 physical activity measures. Both measures were sensitive to change. Because the CHAMPS questionnaire uses response-option categories for duration, analyses were based on the estimated mean minutes per week of physical activity. In contrast, analyses using the BRFSS questions examined percentage of participants meeting physical activity recommendations. Thus, the 2 instruments report different outcomes (minutes vs percentage meeting criterion) and the results, while different, are not necessarily in conflict.

Results were reported separately by program because the programs differed in recruitment strategies, organizational characteristics, types of populations enrolled, length (20 weeks vs 6 months), and mode of delivery. Thus, they could conceivably have different outcome predictors. The findings were similar; both programs generally produced the largest increases in physical activity for participants who were younger and initially less active. The oldest age group may have faced greater chronic and acute health problems and significant life events during the course of the intervention, which may have decreased participation. Although the most active participants made only small increases in physical activity as a result of the program, their level of physical activity was maintained during the 5- to 6-month intervention period.

When we compared program differences, AC produced larger intervention effects among participants with higher levels of pretest physical activity social support. Individuals with low levels of social support may need to be identified at study entry and given additional support or strategies for how to identify and enable support. ALED produced larger intervention gains among women, Latinos/Hispanics, overweight and obese participants, and those with osteoporosis, to the degree that posttest differences in physical activity were eliminated or greatly reduced for these subgroups. These participants may be particularly amenable to this type of instructional group-based approach, which, in contrast to the typical exercise groups offered in many communities, focused on behavioral skills to increase lifestyle physical activity. We are not implying that these types of participants are inappropriate for telephone-supervised home-based programs, because all groups benefited from both programs and posttest differences between programs were modest. The differing populations enrolled in ALED and AC limit the types of direct comparisons and interpretations that can be made.

Predictor analyses can uncover useful findings that inform practice. For example, overweight people assigned to a group-based exercise program in 1 study were the least likely to be successful 2 years later (42). Less educated people who were assigned to a telephone-supervised, home-based exercise program and who were less stressed and less fit at baseline had the greatest probability of success by the second year. As noted earlier, however, few studies have presented these types of analyses.

Several limitations should be considered when interpreting our findings. First, AFL used a quasi-experimental study design with no control group, which prohibited us from conducting true moderator analyses (19) and limited causal inferences. Second, to reduce site and participant burden, we relied on self-reported data. The primary study outcome (physical activity as measured by CHAMPS) correlated moderately with objective physical activity measures, objective measures of physical functioning, and quality of life in other studies (36,37). Third, participants who returned posttest surveys differed from those who did not, and postsurvey response rates were lower than ideal, particularly for the AC program. The evidence base for the types of people who did not return surveys may not be as strong as for those who did. Finally, these types of exploratory analyses should be considered as hypothesis-generating as opposed to hypothesis-testing. Exploratory analyses such as these can identify potential differences in response to the intervention (19). The objective of exploratory research is to develop or refine questions or hypotheses that subsequently require more rigorous testing.

Despite these limitations, this study has a number of strengths, including the large, diverse sample of midlife and older adults and diverse participating community organizations. Relative to the older US population, AFL oversampled African Americans but had similar rates of Latinos and Asians (1). AFL was similar to the older US population in terms of chronic health conditions and health ratings, although our participants were somewhat more likely to report their health as good and somewhat less likely to report it as fair/poor or excellent/very good (43). Participants were more likely to be obese than the older US population (43). Although participants had higher educational levels than the older US population (43), they were less educated than participants in the AC and ALED randomized trials. Our study provides useful information that is generally not reported in the literature regarding predictors of increased physical activity.

In this translational research project, 8 of 17 pretest characteristics were associated with differential outcomes over time. Six of the groups that showed the largest increases in physical activity were least active at pretest, suggesting that the programs worked especially well for those in most need. People older than 75 and those with lower levels of social support at study entry may need more focused or intensive intervention approaches to achieve comparable improvements. Furthermore, longer or more intensive programs may be needed to aid continued increases in physical activity for those who are less educated, less self-efficacious, more stressed, less active, and have more chronic illnesses at program entry.

## Figures and Tables

**Table 1:** Active for Life Lead Organizations, United States, 2003-2006

Lead Organization	Type of Organization
**Active Choices**	

Blue Shield of California (BSC), Woodland Hills, California	Statewide nonprofit health plan
Church Health Center, Memphis, Tennessee	Faith-based health and community development organization
San Mateo County Health Department, San Mateo, California (additional site at the Berkeley Public Health Department, Berkeley, California)	County and city public health departments
YMCA of Metropolitan Chicago, Chicago, Illinois	Nonprofit service organization

**Active Living Every Day**	

Council on Aging of Southwestern Ohio, Cincinnati, Ohio	Aging network organization in partnership with county health district and hospital system
FirstHealth of the Carolinas, Pinehurst, North Carolina	Nonprofit health care delivery system
Greater Detroit Area Health Council, Detroit, Michigan	Regional, membership-based health coalition addressing cost, quality and access to health care
Jewish Council for the Aging of Greater Washington, Rockville, Maryland	Nonprofit human service organization
The OASIS Institute, St. Louis, Missouri (additional sites in Pittsburgh, Pennsylvania, and San Antonio, Texas)	National nonprofit adult learning organization

**Table 2:** Pretest Characteristics of Participants by Program — Active for Life Multisite Study, United States, 2003-2006

**Characteristic**	**Active Choices**(N = 881)	** **Active Living Every Day(N = 1,074)	*P* Value^a^
**Age, y, % **			
50-64	43.7	28.8	<.001
65-74	35.0	37.6	
≥75	21.3	33.6	
**Race/ethnicity, %**			
Non-Hispanic white	41.8	62.8	<.001
Black or African American	36.4	30.4	
Hispanic/Latino	14.5	4.6	
Asian	4.9	0.7	
American Indian/Alaska Native	0.1	0.2	
Reporting 2 groups	1.1	0.4	
Other	0.8	0.6	
Missing	0.3	0.3	
**Sex, %**			
Women	79.1	82.5	.05
Men	20.9	17.5	
**Education level, %**			
No formal education	0.6	0	<.001
Grades 1-8	6.0	2.7	
Grades 9-11	7.2	7.4	
High school/General Educational Development diploma	20.0	28.6	
Some college	29.4	31.5	
College graduate	33.5	29.1	
Missing	3.4	0.7	
**Annual income, $, % **			
<30,000	54.3	46.7	.03
30,000-59,999	24.5	23.6	
≥60,000	15.1	18.3	
Missing	6.1	11.4	
**Marital status, %**			
Married or partnered	40.6	42.7	<.001
Divorced	24.6	17.5	
Widowed	21.5	29.8	
Separated	3.6	1.6	
Never married	8.5	7.9	
Missing	1.1	0.5	
**Body mass index (BMI), mean (SD), kg/m^2^ (n = 1,307)**	30.6 (7.2)	29.5 (6.6)	<.001
**BMI level, %**			
Underweight (≤18.5 kg/m^2^)	0.1	0.8	NA
Normal weight (18.6-24.9 kg/m^2^)	22.2	22.6	
Overweight (25.0-29.9 kg/m^2^)	28.9	33.8	
Obese (≥30 kg/m^2^)	47.0	38.5	
Data missing	1.7	4.2	
**Health conditions, %**			
0-1	45.5	43.4	.33
≥2	54.3	56.5	
Missing	0.2	0.1	
**Participants with chronic conditions, %**			
Diabetes (n = 1,316)	25.2	19.7	.004
Hypertension (n = 1,323)	57.1	56.6	.78
Arthritis (n = 1,325)	54.8	55.4	.98
Coronary heart disease (n = 1,324)	13.2	15.2	.22
Stroke (n = 1,317)	5.3	6.2	.40
Osteoporosis (n = 1,317)	18.4	21.4	.11
**Health rating, %**			
Excellent	4.7	4.5	.21
Very good	19.3	21.7	
Good	48.1	50.5	
Fair	24.0	20.0	
Poor	2.8	2.3	
Missing data	1.1	1.0	
**Psychosocial and behavioral factors, mean (SD)**			
Social support for physical activity (possible range: 5-20) (n = 1,307)	13.7 (3.1)	13.2 (3.0)	.003
Self-efficacy (possible range: 5-35) (n = 1,306)	20.8 (7.1)	19.1 (8.0)	<.001
Depressive symptoms (possible range: 0-30) (n = 1,322)	6.4 (5.2)	5.9 (5.1)	.03
Perceived stress (possible range: 0-16) (n = 1,307)	4.6 (3.1)	4.8 (3.2)	.27
Moderate and vigorous physical activity (CHAMPS), h/wk (n = 1,335)	2.8 (3.9)	2.4 (3.7)	.02
**Physical activity level based on BRFSS physical activity measure, %**			
Sedentary	42.6	42.3	.03
Underactive	45.3	42.3	
Regularly active	10.9	14.9	
Data missing	1.2	0.6	

Abbreviations: BRFSS, Behavioral Risk Factor Surveillance System; CHAMPS, Community Healthy Activities Model Program for Seniors.

a *P* values indicate whether differences in the baseline characteristics of Active Choices and Active Living Every Day participants were significant based on *t* tests for continuous variables and Χ^2^ for categorical variables.

**Table 3:** Adjusted Pretest and Posttest Means for Square Root of Minutes per Week of Moderate-Intensity to Vigorous-Intensity Physical Activity, by Pretest Variables, Active for Life Multisite Study, United States, 2003-2006

Pretest Variable	**Active Choices**							**Active Living Every Day**		

	**Pretest Mean^a^**	**Posttest Mean^a^**	**Effect** size (d)^b^	***P* Cat^c^ (Δ)**	***P*** Contin^d^ (Δ)	**Pretest Mean^a^**	**Posttest Mean^a^**	**Effect** size (d)^b^	***P* Cat^c^ (Δ)**	***P*** Contin **d** (Δ)
**Age, y**										
50-64	9.47	16.48	.81	<.001	<.001	8.78	16.12	.87	<.001	<.001
65-74	10.36	15.82	.59			9.29	15.99	.79		
≥75	9.71	12.78	.38			9.01	12.97	.44		
**Sex**										
Women	8.37	13.85	.64	.55	NA	7.91	14.31	.77	.003	NA
Men	11.05	17.10	.65			1.84	14.74	.40		
**Race/ethnicity**										
White	9.09	14.06	.56	.07	NA	8.30	14.05	.67	.01	NA
Black/African American	10.83	16.33	.62			9.62	15.48	.72		
Hispanic /Latino	8.86	17.20	1.07			7.05	17.70	1.42		
Other	9.70	15.53	.67			10.83	13.26	.26		
**Education**										
Less than high school	8.41	14.58	.79	.66	NA	8.45	13.24	.57	.07	NA
High school/GED	10.31	15.24	.58			9.30	14.39	.61		
More than high school	10.58	16.27	.64			9.95	16.53	.76		
**Body mass index, kg/m^2^**										
<25.0	11.65	16.12	.51	.24	.55	10.22	15.05	.55	.10	.02
25.0-29.9	10.33	16.56	.67			9.82	15.87	.67		
≥30.0	9.64	15.51	.71			7.58	14.29	.86		
**No. of health conditions**										
0-1	10.70	17.09	.71	.07	NA	9.23	14.37	.58	.02	NA
≥2	9.09	14.05	.59			8.39	15.06	.79		
**Diabetes**										
No	10.33	16.14	.66	.46	NA	9.04	14.86	.67	.22	NA
Yes	8.47	13.58	.64			8.59	15.37	.85		
**Hypertension**										
No	9.87	15.39	.62	.83	NA	8.80	14.06	.61	.07	NA
Yes	9.41	15.10	.67			9.22	15.67	.75		
**Arthritis**										
No	9.68	16.02	.73	.09	NA	9.08	14.60	.62	.18	NA
Yes	9.44	14.46	.58			9.05	15.43	.76		
**Coronary heart disease**										
No	9.91	15.83	.68	.07	NA	8.82	14.72	.70	.44	NA
Yes	8.32	12.09	.48			9.66	16.28	.71		
**Osteoporosis**										
No	9.55	15.31	.66	.66	NA	9.48	15.10	.64	.02	NA
Yes	9.58	14.92	.63			7.20	14.64	.92		
**Health rating**										
Fair or poor	7.98	13.95	.80	.59	NA	7.64	14.22	.85	.63	NA
Good	10.03	15.85	.69			8.55	14.36	.69		
Very good or excellent	12.89	17.86	.52			11.21	17.09	.67		
**Social support**										
Lowest 1/3	9.86	14.33	.51	.11	.007	7.78	13.45	.69	.68	.12
Middle 1/3	10.50	15.75	.63			8.34	14.60	.76		
Highest 1/3	10.53	16.95	.71			10.03	16.25	.68		
**Self-efficacy**										
Lowest 1/3	8.69	14.94	.79	.27	.26	6.84	12.80	.83	.80	.75
Middle 1/3	10.49	15.96	.65			9.48	15.70	.74		
Highest 1/3	11.94	16.52	.49			10.64	16.30	.60		
**Depressive symptoms**										
Lowest 1/3	9.75	14.67	.56	.52	.25	8.58	14.84	.71	.09	.46
Middle 1/3	10.31	16.02	.64			9.73	14.84	.58		
Highest 1/3	9.53	15.54	.71			8.86	15.63	.83		
**Perceived stress**										
Lowest 1/3	10.14	14.75	.51	.36	.39	8.25	14.43	.72	.69	.95
Middle 1/3	10.33	16.13	.67			9.35	15.61	.69		
Highest 1/3	10.50	16.38	.68			9.65	15.31	.68		
**Physical activity**										
Lowest 1/3	0.16	10.71	NA^e^	<.001	<.001	0	9.20	NA^e^	<.001	<.001
Middle 1/3	8.64	14.84	2.52			8.03	14.53	2.70		
Highest 1/3	19.32	20.13	.14			19.08	20.50	.26		

Abbreviations:  NA, not applicable; BMI, body mass index.

a Means are adjusted for site clustering, sex, race/ethnicity, education, health rating, and BMI.

b Effect sizes (d = [posttest mean – pretest mean]/pretest standard deviation) use adjusted pretest and posttest means and unadjusted pretest standard deviation. Age, BMI, social support, self-efficacy, depression, stress, and physical activity were treated as continuous variables in change analyses (*P* contin); however, physical activity data and *P* values for the categorical (*P* cat) analyses are also reported to aid interpretations.

c *P* cat (Δ) refers to *P* values for change in physical activity (time x pretest predictor interaction) in instances where the predictor variable was categorical.

d *P* contin (Δ) refers to *P* values for change in physical activity (time x pretest predictor interaction) in instances where the predictor variable was continuous.

e The effect size for the lowest tertile of physical activity could not be computed because the standard deviation (and thus the denominator for the effect size) for that group is zero (ie, all participants in that group reported 0 hours/week of moderate-intensity to vigorous-intensity physical activity).

**Table 4:** Percentage of Active Choices Participants Who Met CDC-ACSM Recommendations (Based on BRFSS Questions) at Pretest and Posttest, by Pretest Variables,^a^ Active for Life Multisite Study, United States, 2003-2006

**Pretest Variable**	Pretest %	Pretest OR(95% CI)	Pretest *P*	Posttest %	Posttest OR(95% CI)	Posttest *P*
**Age, y**						
50-64	10.7	1 [Reference]	.08	35.0	1 [Reference]	.07^b^
65-74	15.3	1.57 (0.90-2.74)		30.9	0.82 (0.51-1.32)	
≥75	9.4	0.86 (0.43-1.73)		21.6	0.49 (0.26-0.91)	
**Sex**						
Women	10.8	1 [Reference]	.44	30.5	1 [Reference]	.59
Men	12.7	1.24 (0.72-2.11)		27.8	0.87 (0.52-1.45)	
**Race/ethnicity**						
Non-Hispanic white	15.1	1 [Reference]	.13	28.2	1 [Reference]	.39
Black/African American	7.7	0.43 (0.21-0.87)		22.0	0.72 (0.41-1.25)	
Hispanic/Latino	10.4	0.71 (0.29-1.73)		30.2	1.15 (0.56-2.34)	
Other	15.1	0.89 (0.39-2.05)		36.2	1.48 (0.67-3.25)	
**Education**						
Less than high school	6.3	1 [Reference]	.05	22.1	1 [Reference]	.21
High school/GED	15.1	5.15 (1.38-19.23)		31.0	1.84 (0.72-4.74)	
More than high school	14.0	4.47 (1.23-16.22)		34.4	2.19 (0.90-5.30)	
**BMI**	NA	0.99 (0.95-1.02)	.48	NA	1.00 (0.97-1.03)	.96
**Health conditions**						
0 to 1	9.6	1 [Reference]	.46	32.1	1 [Reference]	.04^c^
≥2	11.4	1.20 (0.74-1.95)		23.2	0.64 (0.42-0.97)	
**Diabetes**						
No	13.8	1 [Reference]	.08	28.8	1 [Reference]	.72
Yes	9.0	0.56 (0.29-1.06)		30.9	1.10 (0.65, 1.89)	
**Hypertension**						
No	11.5	1 [Reference]	.59	30.0	1 [Reference]	.62
Yes	12.8	1.15 (0.70-1.88)		28.0	0.90 (0.59-1.37)	
**Arthritis**						
No	12.1	1 [Reference]	.82	32.4	1 [Reference]	.03
Yes	12.5	1.06 (0.65-1.71)		23.4	0.63 (0.41-0.95)	
**Coronary heart disease**						
No	11.4	1 [Reference]	.15	31.4	1 [Reference]	.04^c^
Yes	16.0	1.64 (0.84-3.18)		19.6	0.48 (0.23-0.98)	
**Osteoporosis**						
No	11.8	1 [Reference]	.33	29.4	1 [Reference]	.44
Yes	14.9	1.33 (0.75-2.38)		28.3	0.82 (0.49-1.37)	
**Health rating**						
Fair or poor	9.8	1 [Reference]	.33	19.4	1 [Reference]	.04
Good	10.1	1.07 (0.59-1.95)		32.2	2.09 (1.19-3.66)	
Very good or excellent	14.0	1.53 (0.79-2.97)		29.4	1.82 (0.96-3.44)	
**Social support^b^**	NA	1.06 (0.97-1.14)	.19	NA	0.99 (0.93-1.06)	.82
**Self-efficacy^b^**	NA	1.06 (1.02-1.10)	.003	NA	1.03 (1.00-1.06)	.07
**Depressive symptoms^b^**	NA	1.01 (0.96-1.06)	.67	NA	0.99 (0.94-1.03)	.54
**Perceived stress^b^**	NA	1.03 (0.95-1.12)	.43	NA	1.01 (0.94-1.09)	.76
**CHAMPS physical activity^b^**	NA	1.07 (1.04-1.09)	<.001	NA	1.05 (1.02-1.07)	<.001

Abbreviations: CDC-ACSM, Centers for Disease Control and Prevention-American College of Sports Medicine; BRFSS, Behavioral Risk Factor Surveillance System; OR, odds ratio; CI, confidence interval; GED, General Educational Development test; BMI, body mass index; CHAMPS physical activity, Community Healthy Activities Model Program for Seniors physical activity questionnaire.

a All percentages, ORs, and 95% CIs are adjusted for site clustering, sex, race/ethnicity, education, health rating, and BMI. Repeated measures analyses examining pretest to posttest changes (time x pretest predictor interactions) also controlled for site clustering, sex, race/ethnicity, education, health rating, and BMI.

b BMI, social support, self-efficacy, depressive symptoms perceived, stress, and physical activity were treated as continuous variables in change analyses; therefore, percentages that met recommendations are not reported.

c In the repeated measures analyses, a significant time x pretest variable interaction was found (ie, the intervention effect varied by levels of this pretest variable).

**Table 5:** Percentage of Active Living Every Day Participants Who Met CDC-ACSM Recommendations at Pretest and Posttest, by Pretest Variables^a^, Active for Life Multisite Study, 2003-2006

**Pretest Variable**	Pretest %	Pretest OR(95% CI)	Pretest *P*	Posttest %	Posttest OR(95% CI)	Posttest *P*
**Age, y**						
50-64	16.1	1 [Reference]	.40	48.1	1 [Reference]	.36^b^
65-74	19.6	1.35 (0.86-2.12)		43.0	0.81 (0.56-1.17)	
≥75	18.0	1.17 (0.73-1.86)		41.7	0.76 (0.51-1.14)	
**Sex**						
Women	17.6	1 [Reference]	.63	41.2	1 [Reference]	.20
Men	18.2	1.12 (0.71-1.76)		47.3	1.30 (0.87-1.93)	
**Race/ethnicity**						
Non-Hispanic white	13.9	1 [Reference]	.44	36.0	1 [Reference]	.33
Black/African American	14.6	1.08 (0.63-1.86)		37.9	1.09 (0.72-1.63)	
Hispanic/Latino	17.9	1.48 (0.61-3.60)		46.5	1.55 (0.73-3.29)	
Other	25.2	2.28 (0.73-7.15)		56.8	2.40 (0.77-7.46)	
**Education**						
Less than high school	18.6	1 [Reference]	.68	35.0	1 [Reference]	.04^b^
High school/GED	18.1	0.86 (0.42-1.73)		46.1	1.71 (0.90-3.24)	
More than high school	17.0	0.76 (0.39-1.51)		51.7	2.16 (1.15-4.05)	
**BMI^c^**	NA	0.93 (0.90-0.96)	<.001	NA	0.98 (0.96-1.01)	.13^b^
**Health conditions**						
0 to 1	19.5	1 [Reference]	.20	49.3	1 [Reference]	.15
≥2	17.2	0.78 (0.53-1.14)		43.9	0.80 (0.59-1.09)	
**Diabetes**						
No	17.3	1 [Reference]	.63	44.9	1 [Reference]	.73
Yes	16.0	0.89 (0.54-1.46)		46.6	1.07 (0.71-1.61)	
**Hypertension**						
No	16.7	1 [Reference]	.54	45.6	1 [Reference]	.68
Yes	18.3	1.12 (0.78-1.60)		44.0	0.94 (0.68-1.28)	
**Arthritis**						
No	18.8	1 [Reference]	.46	43.4	1 [Reference]	.78
Yes	17.0	0.88 (0.62-1.45)		44.9	1.05 (0.76-1.45)	
**Coronary heart disease**						
No	17.2	1 [Reference]	.19	44.8	1 [Reference]	.72
Yes	21.3	1.38 (0.85-2.25)		43.0	0.92 (0.57-1.47)	
**Osteoporosis**						
No	18.2	1 [Reference]	.50	44.2	1 [Reference]	.87
Yes	16.9	0.87 (0.57-1.31)		45.0	1.03 (0.70-1.52)	
**Health rating**						
Fair or poor	16.8	1 [Reference]	.01	44.9	1 [Reference]	.24
Good	16.5	1.02 (0.60-1.71)		44.4	0.98 (0.66-1.46)	
Very good or excellent	24.9	1.80 (1.04-3.09)		51.6	1.32 (0.83-2.09)	
**Social support^c^**	NA	1.10 (1.04-1.17)	.06	NA	1.05 (1.00-1.11)	.05
**Self-efficacy^c^**	NA	1.04 (1.01-1.06)	.003	NA	1.04 (1.02-1.06)	<.001
**Depressive symptoms^c^**	NA	0.98 (0.94-1.02)	.38	NA	0.99 (0.96-1.03)	.74
**Perceived stress^c^**	NA	0.95 (0.90-1.01)	.11	NA	0.93 (0.88-0.98)	.01
**CHAMPS physical activity^c^**	NA	1.10 (1.08-1.12)	<.001	NA	1.05 (1.03-1.07)	<.001^b^

Abbreviations: CDC-ACSM, Centers for Disease Control and Prevention-American College of Sports Medicine; OR, odds ratio; CI, confidence interval; GED, General Educational Development test; BMI, body mass index; CHAMPS physical activity, Community Healthy Activities Model Program for Seniors physical activity questionnaire.

a All percentages, ORs, and 95% CIs are adjusted for site clustering, sex, race/ethnicity, education, health rating, and BMI. Repeated measures analyses examining pretest to posttest changes (time x pretest predictor interactions) also controlled for site clustering, sex, race/ethnicity, education, health rating, and BMI.

b In the repeated measures analyses, a significant time x pretest variable interaction was found (ie, the intervention effect varied by levels of this pretest variable).

c BMI, social support, self-efficacy, depressive symptoms, perceived stress, and physical activity were treated as continuous variables in change analyses; therefore, percentages that met recommendations are not reported.
